# Measuring physical activity during pregnancy

**DOI:** 10.1186/1479-5868-8-19

**Published:** 2011-03-21

**Authors:** Cheryce L Harrison, Russell G Thompson, Helena J Teede, Catherine B Lombard

**Affiliations:** 1 Jean Hailes Foundation for Women's Health Research Unit, School of Public Health and Preventive Medicine, Monash University, Clayton, Victoria, Australia; 2 Diabetes Unit, Southern Health, Clayton, Victoria, Australia; 3 Institute of Transport Studies, Monash University, Clayton, Victoria, Australia

## Abstract

**Background:**

Currently, little is known about physical activity patterns in pregnancy with prior estimates predominantly based on subjective assessment measures that are prone to error. Given the increasing obesity rates and the importance of physical activity in pregnancy, we evaluated the relationship and agreement between subjective and objective physical activity assessment tools to inform researchers and clinicians on optimal assessment of physical activity in pregnancy.

**Methods:**

48 pregnant women between 26-28 weeks gestation were recruited. The Yamax pedometer and Actigraph accelerometer were worn for 5-7 days under free living conditions and thereafter the International Physical Activity Questionnaire (IPAQ) was completed. IPAQ and pedometer estimates of activity were compared to the more robust and accurate accelerometer data.

**Results:**

Of 48 women recruited, 30 women completed the study (mean age: 33.6 ± 4.7 years; mean BMI: 31.2 ± 5.1 kg/m^2^) and 18 were excluded (failure to wear [n = 8] and incomplete data [n = 10]). The accelerometer and pedometer correlated significantly on estimation of daily steps (ρ = 0.69, *p *< 0.01) and had good absolute agreement with low systematic error (mean difference: 505 ± 1498 steps/day). Accelerometer and IPAQ estimates of total, light and moderate Metabolic Equivalent minutes/day (MET min^-1 ^day^-1^) were not significantly correlated and there was poor absolute agreement. Relative to the accelerometer, the IPAQ under predicted daily total METs (105.76 ± 259.13 min^-1 ^day^-1^) and light METs (255.55 ± 128.41 min^-1 ^day^-1^) and over predicted moderate METs (-112.25 ± 166.41 min^-1 ^day^-1^).

**Conclusion:**

Compared with the accelerometer, the pedometer appears to provide a reliable estimate of physical activity in pregnancy, whereas the subjective IPAQ measure performed less accurately in this setting. Future research measuring activity in pregnancy should optimally encompass objective measures of physical activity.

**Trial Registration:**

Australian New Zealand Clinical Trial Registry Number: ACTRN12608000233325. Registered 7/5/2008.

## Background

Regular physical activity plays a fundamental role in health and is positively associated with a reduced risk of obesity, hypertension, diabetes and cardiovascular disease (CVD) and a variety of other health conditions. Additional advantages of regular physical activity apply to specific settings, such as pregnancy, with benefits including improved emotional well being and body image and reduced risk of gestational diabetes mellitus[1-3], excess maternal weight gain [4] and complications during labour [5]. Exercise during pregnancy also has positive effects on the foetus including improved stress response in utero and reduced risk of childhood obesity [6]. In the presence of limited pregnancy specific research, current activity recommendations are informed by general advice for healthy adults [7]. In the absence of any contraindications, pregnant women are advised to engage in physical activity of moderate intensity and duration (~30 minutes) on most days of the week [7].

Despite the potential health benefits, there is limited accurate information about physical activity patterns during pregnancy [8]. Previous epidemiological research suggests that most women (~50-60%) do not participate in regular physical activity during pregnancy [9,10]. However, these estimates are based around use of crude measures that are not validated [10] and may be prone to error [11]. A recent review highlighted the inadequacy of previous research in measuring physical activity during pregnancy, with studies predominantly using subjective long-term, self-recall of activity with no published evidence of their reliability or validity [11]. Further, there are limited pregnancy specific studies that have objectively assessed physical activity with pedometers [8,12-14] or accelerometers [15,16]. Given the limited research using objective, comprehensive and validated methods, there is currently no commonly accepted measurement tool used to assess physical activity during pregnancy.

In non-pregnant populations, the gold standard measurement of free-living energy expenditure is the use of doubly labelled water (DLW) [17] which can also be used to estimate activity related energy expenditure. However, DLW is expensive, time consuming, requires experienced operators and therefore other, more feasible tools are generally used to estimate physical activity in population studies [17]. The accelerometer is an alternative objective tool that is both comprehensive and validated against the gold standard DLW technique in non pregnant populations [17]. Given the complexity, cost and expertise required to use the DLW technique, the accelerometer may be more feasible in community based research settings. In addition to measuring step count, the accelerometer measures duration, frequency and intensity of activity, thereby providing accurate information on physical activity in free-living conditions [17].

Subjective, self-recalled measures, including physical activity recall questionnaires are widely used in epidemiological studies being simple and low in cost. The International Physical Activity Questionnaire (IPAQ) [18] is a standardised questionnaire widely used to assess walking, moderate and vigorous activity across habitual domains, including leisure time. IPAQ has previously been validated using the Actigraph accelerometer for estimating physical activity in non-pregnant populations [19]. In the validation study (n = 2721), a fair correlation between the IPAQ (long version) and accelerometer was reported (r_s _pooled = 0.33), however no assessment of absolute agreement was performed between measures [19]. In previous pregnancy related studies the IPAQ has been applied [20] with validity and accuracy unknown in this setting [11]. Earlier studies have raised concerns over self-recalled questionnaires in pregnancy, which may be insensitive in capturing low intensity activities including walking [10]. Walking is the most popular form of activity, however accurate recall is difficult compared to structured or vigorous activity [10-12,21]. Pedometers, a popular and widely accepted objective measurement tool, estimate total steps and distance and are a validated and reliable method for assessing physical activity in non-pregnant populations [22,23]. Although pedometers have been applied in pregnancy, there are theoretical concerns about the body shape changes in pregnancy. Therefore the role of pedometers in pregnancy remains unclear [8]. With accuracy of most popular subjective and objective tools unknown in pregnancy, there is a need to evaluate these against comprehensive techniques, including accelerometers, to inform on optimal evaluation of physical activity during pregnancy.

In this setting, we aimed to evaluate the relationship and agreement between the accelerometer and subjective (IPAQ) and objective (pedometer) physical activity measurement tools in pregnancy. As the second trimester is considered the most comfortable period during pregnancy [12], we studied women between 26-28 weeks gestation consistent with previous research [8,12,15].

## Methods

### Subjects

**Figure 1:**
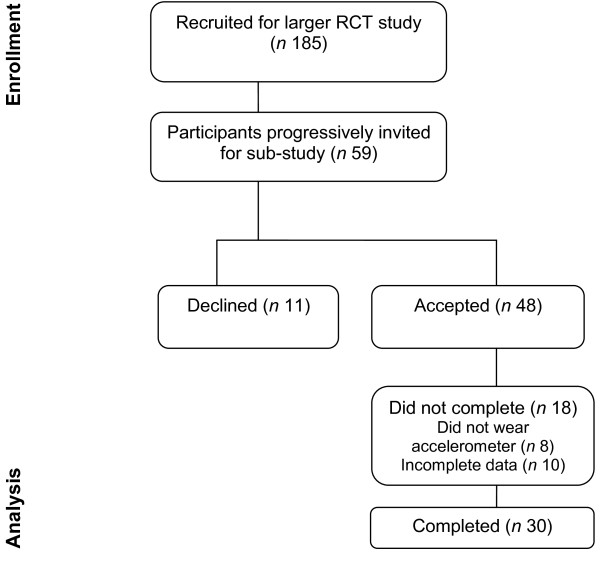
**CONSORT criteria for included participants**.

This study involved a subset of women recruited for a larger randomised controlled trial (RCT) promoting healthy living (intervention to improve diet and physical activity) in pregnancy versus standard information only (controls) from early pregnancy (13-16 weeks gestation) to six weeks postpartum. A flow diagram of participants, according to the CONSORT statement, are available in Figure 1[24]. Women were recruited for the larger RCT study from May 2008 onwards through invitation flyers given at the first maternal appointment in early pregnancy at a large tertiary hospital in Australia. Inclusion criteria for the larger RCT study included women who were overweight (Body Mass Index; BMI ≥ 25.0 kg/m^2^) and experiencing a singleton pregnancy. Exclusion criteria included a BMI ≥ 46 kg/m^2 ^and pre-existing, ongoing medical conditions. Group allocation was achieved through computer generated randomised sequencing. Researchers were blinded to group allocation through envelope concealment at point of recruitment.

For the sub-study, recruitment took place over a 12 month period (i.e. March 2009-March 2010). Women recruited into the larger RCT study who were between 26-28 weeks of pregnancy during this 12 month period were sequentially invited, according to their study identification number, to participate until adequate numbers were reached. There were equal numbers of participants for the accelerometer sub-study, representative from both study groups (i.e. control and intervention). As the sub-study involved the same protocol and measures for all participants, no additional randomisation or allocation was required. The Southern Health Research Advisory and Ethics Committee approved the study and all participants gave written informed consent.

### Measures

#### International Physical Activity Questionnaire (IPAQ) long version

The IPAQ was used to assess physical activity across a variety of different domains including leisure-time, domestic, work and transport related physical activity [18,19]. Each domain assesses walking, moderate and vigorous physical activity performed for at least 10 consecutive minutes each day, over seven days. An average metabolic equivalent (MET) score was calculated for total physical activity performed per week as a continuous variable whereby total physical activity in MET-minutes/week = sum of total [Walking + Moderate + Vigorous] MET minutes/week scores. Individual MET scores for walking, moderate and vigorous activity were calculated within each domain and combined to provide a total score using the following equations: total MET-minutes/week = Met-level × minutes per day × days per week, where 1 MET is equivalent to resting energy expenditure.

#### Pedometer

The Yamax Digiwalker SW-700 Pedometer (Yamax Corporation, Tokyo, Japan) was used to assess the number of free-living steps per day as a valid and reliable tool to measure step count in free living and controlled conditions in non pregnant populations [22,25,26]. The Yamax pedometer is spring-levered and detects vertical plane movement when a force of ≥0.35*g *is registered [27]. The pedometer was sealed to blind participants and limit motivation to increase walking as previously reported [28]. The pedometers were programmed to an average stride length of 50 cm. Pedometer readings were accumulated for a minimum of five and up to seven consecutive days including at least one weekend day, during waking hours (excluding water activities) which has previously shown to be sufficient for estimating weekly physical activity in women [29].

#### Accelerometer

The Actigraph GT1 M (Actigraph, Florida, US) assesses total body displacement across a vertical plane and determines physical activity levels in the form of activity and step counts. A step is registered with a force threshold of ≥0.30*g *and intensity of movement is determined by magnitude of force in increments of 0.05*g *up to 2.0*g *[27]. Activity counts, recorded every minute, were summed to provide total counts per day and converted to METs in specific intensity levels of light (100-1952 counts*min^-1^), moderate (1953-5724 counts*min^-1^) and vigorous (> 5725 counts*min^-1^) according to the protocol adopted by Freedson et al (1998) [30]. A threshold of <100 counts*min^-1 ^was used to define sedentary behaviour as used previously [19,31,32]. In conjunction with the pedometer, accelerometer readings were accumulated for a minimum of five and up to seven consecutive days including at least one weekend day, during waking hours (excluding water activities). The Actigraph accelerometer has previously gained validity and reliability in free living conditions in adults for between three to five days [33].

#### Demographics and Anthropometric Information

Participants commenced the main RCT study between 13-16 weeks gestation at which time all baseline measurements were assessed. At baseline, demographic information was collected in addition to anthropometric assessment including weight on an electronic scale, calibrated biannually, to the nearest 0.1 kg (Tanita model BWB-800 Digital Scale, Wedderburn Scales, Melbourne, Australia) and height performed by a registered nurse unaware of participant allocation. At 26-28 weeks gestation women recruited for the sub-study were weighed prior to distributing the pedometer, accelerometer and the IPAQ activity questionnaire.

### Procedure

Invited women that agreed to participate in this sub-study (*n *48) were provided with the accelerometer to which the pedometer was then attached adjacent on an adjustable elastic strap. To reduce tilt, the elastic strap was worn around the bottom of the stomach with participants instructed to wear the devices on the hip in line with the left or right knee according to standard instructions. All participants were provided with an information sheet containing pictures and text on correct usage. A full day was considered as wearing the devices for at least eight daytime hours and a half day was considered as less than eight hours but more than three hours. If worn for less than three hours a day, this was treated as a missing day. A simple exercise diary was provided in which participants were instructed to record daily when they put on and took off the devices. Participants were also provided with a long version of the IPAQ and instructed to complete it following the five to seven days of wearing both devices.

### Statistics

All data collected was analysed using SPSS Data Analysis version 19.0 (SPSS Inc, Chicago, IL) in collaboration with a senior biostatistician. As the duration of wearing the pedometer and accelerometer varied between five to seven days between participants and MET min^-1 ^calculated by IPAQ provide a total value for seven full days, data collected was processed to report average daily steps or MET min^-1^. As physical activity data were not evenly distributed, Wilcoxon signed-rank tests were used to compare median differences between methods used. Spearman correlation was used to assess the relationship between estimates of activity measured, including daily step count and daily total, light and moderate MET min^-1^. Regression and Bland-Altman analysis were used to assess the agreement between the MET min^-1 ^day^-1 ^estimated by the accelerometer-IPAQ and steps estimated by the accelerometer-pedometer in assessing physical activity. Bland-Altman analysis uses mean differences between devices and does not assume normality [34,35]. A power analysis was performed to determine the minimal sample size required in order to detect a correlation coefficient of 0.5 between two measures. In order to achieve at least 80% power with a significance level of α = 0.05, a sample size of *n *= 30 was required. All results are presented as mean (SD), unless otherwise stated.

## Results

### Demographic Characteristics

Of the 48 participants recruited into the sub study, a total of 30 completed all requirements for the study and provided complete data sets. 18 women were excluded due to not wearing the devices (n = 8; i.e. due to discomfort, forgot to wear or change of mind in participation) or incomplete data (n = 10; i.e. did not wear both devices in conjunction for a minimum of five days; Figure 1). The mean age and BMI of participants at point of recruitment, between 13-16 weeks gestation was 33.6(±4.7) years and 31.2(±5.1) kg/m^2 ^respectively. At 26-28 weeks gestation, average BMI was 33.6(±4.6) kg/m^2^. Demographic characteristics are presented in Table 1. The majority of participants were born in Australia (53.3%) and working at least part-time (56.7%) with a household income of at least $40,000 AUD (46.7%). The majority of participants were experiencing either their first or second pregnancy (73.3%).

**Table 1:** Demographic characteristics of participants (*n *= 30).

Variable	*n (*%)
*Country Of Birth*	
Australia	16 (53)
Other	14 (47)
*Education*	
Year 10-11	3 (10)
Year 12	3 (10)
Certificate/diploma	8 (27)
University degree or higher	16 (55)
*Work*	
Full Time	7 (23)
Part Time	10 (33)
No paid work	13 (43)
*Occupation (n 17)*	
Professional	3 (18)
Manager	5 (29)
Clerical	9 (53)
*Income ($)*	
<40,000	9 (30)
40-60,000	2 (7)
60-80,000	5 (17)
>80,000	7 (23)
Unsure/Declined	7 (23)
Previous Pregnancies	
One	11(37)
Two-five	8 (27)
First pregnancy	11(37)

### Relationship and agreement between subjective and objective activity measurement tools

**Figure 2:**
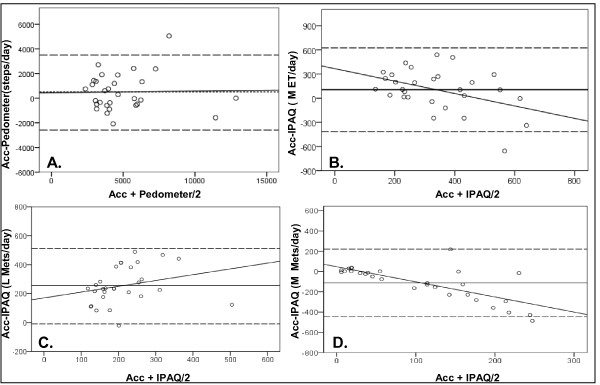
**Bland-Altman plots of physical activity measurement agreement between the Actigraph accelerometer and pedometer steps (A); Actigraph accelerometer and IPAQ mean daily total MET min^-1 ^(B); light MET min^-1 ^(C) and moderate MET min^-1 ^(D)**.

Participants wore both devices for an average of 6.5(±0.8) days for 10.9(±1.9) hours per day. Mean daily steps estimated by the pedometer and accelerometer and daily MET min^-1 ^estimated by the accelerometer and IPAQ are presented in Table 2. There was a moderately strong correlation between the accelerometer and pedometer for estimating daily steps (ρ = 0.69, *p *< 0.01) and good absolute agreement as indicated by low systematic error (r^2 ^= 0.0004) and no significant difference between devices in estimating daily steps (*p *= 0.14). There was a mean difference between devices of 505 ± 1498 steps/day (Figure 2). The limits of agreement, as displayed in the Bland-Altman analysis were -2491 to 3501 steps/day. There was also a fair relationship between the mean daily pedometer steps and accelerometer total MET min^-1 ^(ρ = 0.57, *p *< 0.01) and light MET min^-1 ^(ρ = 0.41, *p *< 0.05). There was a weak relationship between pedometer steps and accelerometer mean daily moderate MET min^-1^, however this was not significant (ρ = 0.34, p = 0.06).

**Table 2:** Daily estimates by pedometer, accelerometer and IPAQ.

Variable	Mean (SD)
*Pedometer*	
Total steps	4679.85 (2520.04)
*Accelerometer*	
Total steps	5185.43 (2550.66)
Total MET min^-1^	403.10 (158.40)
Light MET min^-1^	352.75 (133.76)
Moderate MET min^-1^	50.51 (59.10)
*IPAQ*	
Total MET min^-1^	285.29 (230.96)*
Light/Walk MET min^-1^	85.02 (91.18)**
Moderate MET's	162.93 (153.90)**

* Significantly different from accelerometer MET min^-1 ^estimates (*p *< 0.05) and ** (*p *< 0.01)

Accelerometer and IPAQ estimates of total MET min^-1 ^day^-1 ^(ρ = 0.15, *p *= 0.44), light MET min^-1 ^day^-1 ^(ρ = 0.03, *p *= 0.86) and moderate MET min^-1 ^day^-1 ^(ρ = 0.09, *p *= 0.66) were not significantly correlated and significant differences were found between all estimates of mean MET min^-1 ^day^-1 ^as depicted in Table 2. There was poor absolute agreement and systematic error between the accelerometer and IPAQ for the estimation of average daily total (r^2 ^= 0.18, *p *< 0.01), light (r^2 ^= 0.07, *p *< 0.01) and moderate (r^2 ^= 0.54, *p *< 0.01) MET min^-1 ^(Figure 2). Relative to the accelerometer, the IPAQ under predicted daily total MET min^-1 ^(105.76 ± 259.13 min^-1 ^day^-1^) and light MET min^-1 ^(255.55 ± 128.41 min^-1 ^day^-1^) and over predicted moderate MET min^-1 ^(-112.25 ± 166.41 min^-1 ^day^-1^). The limits of agreement in the Bland Altman analysis were considerably wide for all three comparisons including daily total MET min^-1 ^(-412 to 624 MET min^-1 ^day^-1^), light MET min^-1 ^(-10 to 511 MET min^-1 ^day^-1^) and moderate MET min^-1 ^(-445 to 220 MET min^-1 ^day^-1^; Figure 2). Additionally, there was no relationship between mean IPAQ daily MET min^-1 ^and accelerometer steps (ρ = 0.17, p = 0.38) or pedometer steps (ρ = 0.30, p = 0.12; Table 2). Vigorous activity was not detected or self-reported by the accelerometer or in the IPAQ, respectively and therefore was not included in results.

## Discussion

The purpose of this study was to assess the performance of simple objective (pedometer) and subjective (IPAQ) physical activity measurement tools against the comprehensive objective accelerometer in pregnancy. The results of the current study show that relative to the accelerometer, the pedometer provided better estimates of physical activity in pregnancy than the IPAQ, which showed poor absolute agreement and systematic error. The pedometer showed moderately strong relative agreement (ρ = 0.69) and good absolute agreement with the accelerometer, only slightly underestimating daily steps (505 steps). The mean daily steps estimated by the pedometer also correlated well against mean daily total (ρ = 0.57) and categorised light MET min^-1 ^(ρ = 0.41) estimated by the accelerometer. In comparison, the IPAQ showed no relationship (ρ = 0.15) and poor agreement with the accelerometer for estimating daily physical activity, calculated in MET min^-1^, which persisted even when MET min^-1 ^were further categorised in to light and moderate activity indices. Additionally, no relationship was observed between accelerometer and pedometer estimated steps and IPAQ estimated mean daily total MET min^-1 ^(ρ = 0.17 and 0.30, respectively). Consistent with our findings, other non-pregnant studies reporting relationships between subjective and objective measures have also shown weaker relationships in comparison to relationships between objective measures [36].

This is the first study to apply robust techniques, including assessment of agreeability, to compare physical activity measures in pregnancy. To date, very few studies have used objective measures to assess activity levels in pregnancy [8,12-16], with most relying on less reliable subjective self-report measurement tools. With accuracy of self-reported methods unknown in pregnancy, previous studies using these methods may be misleading. To our knowledge, there are no prior pregnancy specific studies that assess agreeability between physical activity estimates. Although strength of relationship can be useful, correlation analysis does not assess whether two methods agree or differ from each other [34,37]. Indeed, methods measuring the same outcome would be expected to have a strong relationship. As there is no accepted measure of physical activity in pregnancy, there was a need to assess common and widely available measures against more comprehensive techniques to assess both agreeability and accuracy.

Our results are consistent with data from non-pregnant populations, where strong relationships have also been reported between the accelerometer and pedometer [27,36,38-40] with high agreeability [36] or small mean differences between devices [40]. Generally, consistent with our findings, the pedometer appears to under-estimate total step count by approximately 10% based on accelerometer data [40]. Contributors to this may include the increased force required to register a step with the pedometer, especially at lower speeds [27,39,40] and increased tilt angle on the abdominal or hip area that may occur in pregnancy [12]. Additionally, as the force required to register a step is lower in the accelerometer, more nonambulatory movements are likely to be registered as steps including lifting, twisting and bending, potentially overestimating steps [41]. Taken together, these factors may account for the small difference in steps measured by the two instruments. To increase accuracy, using instruments with the same force threshold may correct discrepancies between instruments [41].

The results of the current study also confirm limited previous research suggesting poor correlations between pedometers and subjective measures of physical activity in pregnancy. In a study estimating activity with the leisure time exercise questionnaire (LTEQ) and the pedometer at 20 weeks gestation, no relationship was observed for total (r^2 ^= 0.24) or mild (r^2 ^= 0.13) LTEQ physical activity minutes per week, although a fair correlation was reported for moderate activity minutes (r^2 ^= 0.35, *p <*0.05) in comparison to the pedometer [12]. With pregnant women likely to participate less in physical activity during pregnancy this may contribute to a reduced ability to accurately recall activity over long periods of time. Further, overreporting and underreporting is common in physical activity recall questionnaires, including the IPAQ [42-44], with accuracy particularly reduced when walking is estimated [42]. Therefore, in pregnancy where walking is more likely in comparison to other activities [45], subjective measures may not be ideal. These considerations may, in part, explain the lack of relationship found between subjective and objective measures found here and in previous studies. Other studies comparing self-reported diaries and pedometers have reported fair (r^2 ^= 0.49, *p *< 0.02) [8] to strong (r^2 ^= 0.76, *p *< 0.01) [15] correlations between measures in pregnant women at 20-28 weeks gestation. Frequent recording of physical activity [15] is more intensive and may contribute to increased awareness of physical activity, increasing the likelihood of stronger relationships between subjective and objective methods, even if activity levels are low. Therefore, despite their intensity, future studies assessing physical activity levels subjectively in pregnancy may benefit from using self-report diaries to increase likelihood of more accurate results, rather long-term recall questionnaires.

The IPAQ was chosen in this study as the questionnaire attempts to estimate mild, moderate and vigorous physical activity across a number of different domains, including activates that may be especially important during pregnancy (i.e. child care, household duties) [46]. However, despite the added domains in the IPAQ, our results suggest it still is insensitive in capturing physical activity during pregnancy. Alternatively, the added domains within IPAQ may increase inaccuracy in self-reporting with participants required to interpret and separate activity in to specific domains. These considerations highlight the need for an effective, yet simple validated self-recalled measure that accurately captures physical activity levels during this time. Although this study demonstrated the accuracy of pedometer estimates of activity levels in pregnancy, future research would benefit by conducting validation studies for objective measurement tools including the pedometer in pregnancy, as validity was not assessed in the current study.

There are limitations in the current study. As the aim of study was to evaluate activity tools in pregnancy, the results found are specific for pregnancy and may not be easily generalised to other non-pregnant populations. There are a number of popular self-recalled methods developed for the general population [47] and to a lesser extent for pregnancy [48] however we assessed the IPAQ due to wide acceptance and validity in previous non-pregnant studies [19] and it is possible that use of an alternative self-recall method may have produced different results. Additionally, although we provided detailed instructions on correct usage, ~37% of participants recruited were excluded due to incomplete data or not wearing devices, indicating that some participants may have experienced difficulty in wearing the objective devices. A limitation of both the pedometer and accelerometer is their ability to tilt potentially contributing to altered recordings, including missing steps and conversely, nonambulatory movements registering as steps. Additionally, both devices are unable to measure and record water based activities exercises that may become more popular during pregnancy. Strengths include robust statistical techniques and recruiting a representative group of women from diverse backgrounds, with approximately 50% born in Australia and the remaining 50% born in countries other than Australia with differing household incomes and working arrangements.

## Conclusions

In summary, this novel and comprehensive evaluation of physical activity measurement in pregnancy has demonstrated that in comparison to the subjective IPAQ, the objective pedometer has high relative and absolute agreement with the comprehensive accelerometer tool. Whilst further research on other subjective measures of physical activity is warranted, researchers should consider using the widely available pedometer as an accurate measure of physical activity in pregnancy. As current knowledge and recommendations about physical activity levels in pregnancy are based on self-recalled methods which may be less accurate, we propose that further research incorporating objective measures of physical activity is important to inform clinicians, community and policy makers on optimal physical activity recommendations in pregnancy.

## Conflict of Interest Statement

The authors declare that they have no competing interests. CH is a National Health and Medical Research (NHMRC) public health PhD scholar. HT is an NHMRC research fellow.

## Authors' contributions

CLH conceptualised and designed the research study, recruited participants, collected and analysed data, performed statistical analysis and drafted the manuscript. RGT conceptualised the study, performed accelerometer data analysis and approved the manuscript for publication. HJT designed the research study, interpreted the results, reviewed the manuscript for intellectual content and approved the manuscript for publication. CBL designed the study, interpreted the results, reviewed the manuscript for intellectual content and approved the manuscript for publication.
